# The multistep process of vaginal cancer arising from deep infiltrating endometriosis: a case report

**DOI:** 10.1186/s12905-021-01410-5

**Published:** 2021-07-12

**Authors:** Jee Hyun Kim, Seung Hun Song, Gwangil Kim, Kyoung Ah Kim, Woo Ram Kim

**Affiliations:** 1 Department of Fertility Center, CHA Bundang Medical Center, CHA University School of Medicine, Seongnam, South Korea; 2 Department of Comprehensive Gynecologic Cancer Center, CHA Bundang Medical Center, CHA University School of Medicine, Seongnam-si, Gyeonggi-do 13496 South Korea; 3 Department of Pathology, CHA Bundang Medical Center, CHA University School of Medicine, Seongnam, South Korea; 4 Department of Radiology, CHA Bundang Medical Center, CHA University School of Medicine, Seongnam, South Korea; 5 Department of Surgery, CHA Bundang Medical Center, CHA University School of Medicine, Seongnam, South Korea

**Keywords:** Deep infiltrating endometriosis, Vaginal cancer originating from endometriosis, Synchronous endometrial cancer, Endometriosis-associated complex hyperplasia, Endometriosis-associated endometrioid cancer

## Abstract

**Background:**

Malignant transformation of endometriosis in extraovarian sites remains rare. Furthermore, the process is not definitely understood.

**Case presentation:**

Herein, we report the case of a 40-year-old premenopausal nulligravida woman who presented with vaginal bleeding and who was finally diagnosed with a vaginal cancer originating from endometriosis and with a synchronous endometrial cancer. A gynecologic examination revealed a multiple polypoid mass on the posterior vaginal fornix. Magnetic Resonance Imaging of the pelvis showed two masses abutting respectively on the anterior uterine wall, and in the rectovaginal septum. The patient underwent a total laparoscopic excision of the rectovaginal mass, radical hysterectomy and low anterior resection of the rectum. The lesions were diagnosed as endometriosis, endometriosis-associated complex hyperplasia and endometrioid cancer. Furthermore, a synchronous endometrioid endometrial cancer was reported.

**Conclusions:**

This case revealed the multistep process of malignant transformation of deep infiltrating endometriosis. The progression was individualized between implantation sites and in the same organ.

## Background

Endometriosis is defined as the abnormal presence of endometrial tissue, most often on the ovaries, fallopian tubes, around the ureters, and more rarely in extrapelvic locations, such as bowel, rectum, and bladder [1–3]. It can rarely be presenting as vaginal mass and vaginal endometriosis is difficult to diagnosis [4, 5]. Endometriosis is considered a gynecological benign condition. However, malignant transformation of endometriosis occurs in less than 1% of endometriosis cases, and 78.7% of them had ovarian endometriosis transforming into clear cell or endometrioid ovarian cancer [6]. Recently, the malignant process of abdominal wall endometriosis which was transformed to clear cell borderline tumor has been reported [7], while malignant transformation of deep infiltrating endometriosis has been rarely reported [8], however, the malignant process is not yet well understood.

Endometriosis is associated with the risk of endometrial cancer [9, 10]. Since the hormonal milieu may influence the pathogenesis of these two diseases, women with endometriosis have an increased risk of subsequent endometrial cancer [11]. When endometriosis-associated cancer and endometrial cancer coexist, the differential diagnosis to determine whether these diseases are double primary cancers or metastatic cancer from each can be challenging.

Herein, we report the case of a 40-year-old woman diagnosed with vaginal cancer originating from endometriosis and synchronous endometrial cancer.

## Case presentation

A 40-year-old premenopausal nulligravida woman presented with vaginal bleeding in January 2020. The patient was otherwise well, had no prior surgery, and was not taking hormone medication. Her medical history was significant for morbid obesity, with a body mass index (BMI) of 36.4 kg/m^2^.

**Fig. 1:**
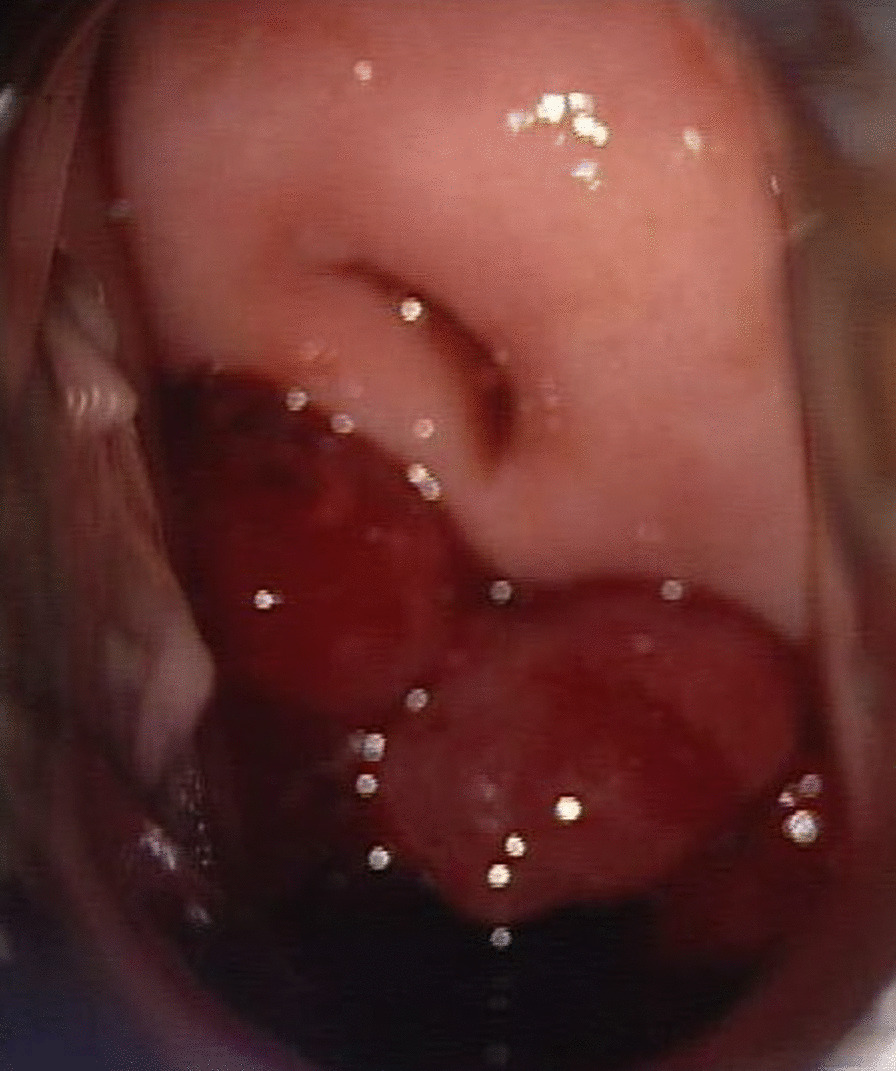
Multiple polypoid masses on the posterior vaginal fornix

**Fig. 2:**
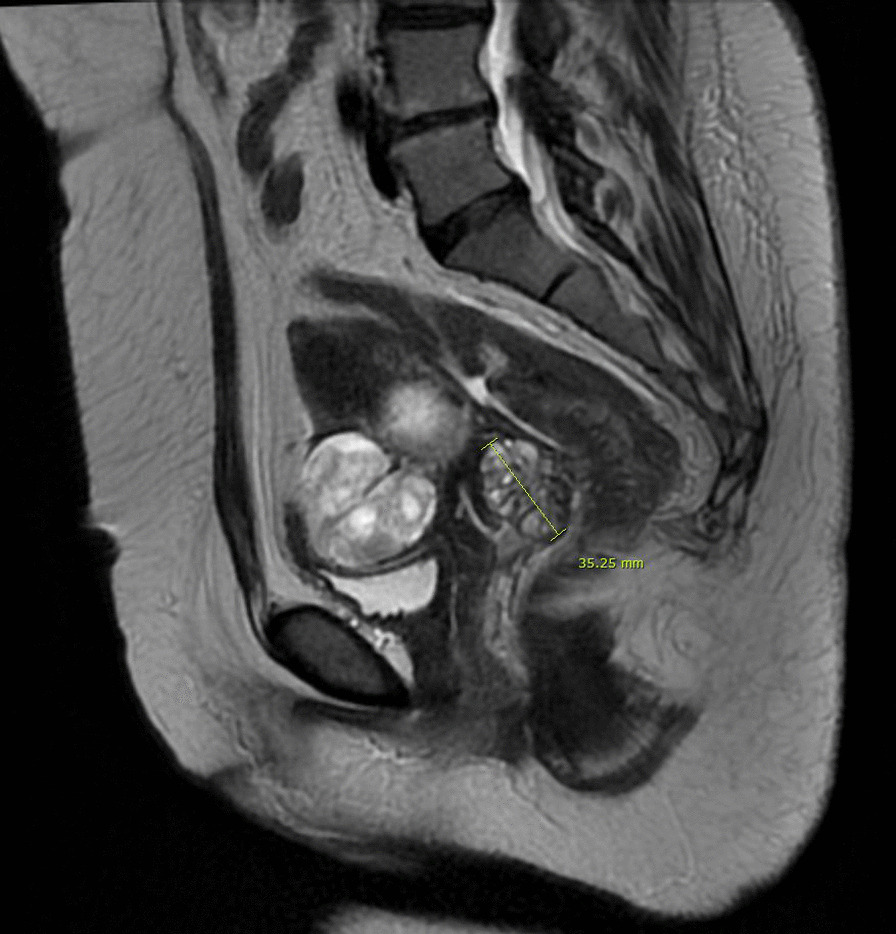
Magnetic Resonance Imaging of the pelvis finding of a rectovaginal mass which invades vaginal and rectal wall

**Fig. 3:**
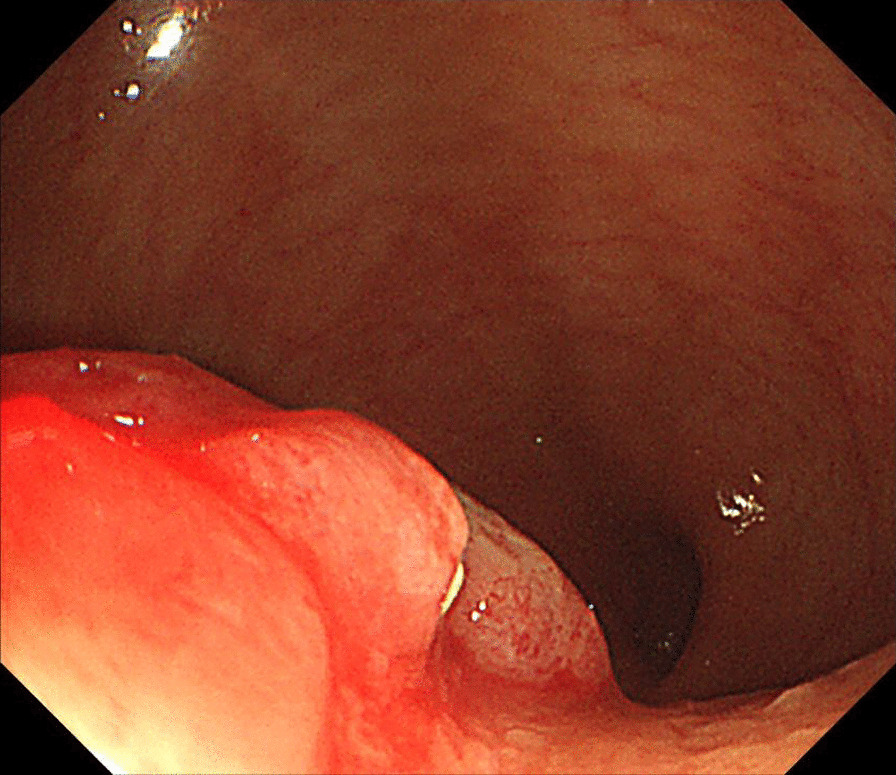
Sigmoidoscopic finding of rectal endometriosis

A gynecologic examination revealed a multiple polypoid mass on the posterior vaginal fornix. (Fig. 1). Punch biopsy revealed endometriosis. The cervix appeared normal. A cervical smear showed reactive cellular changes but was negative for human papilloma virus. Magnetic Resonance Imaging of the pelvis showed two masses abutting respectively on the anterior uterine wall, and in the rectovaginal septum. (Fig. 2), diffuse thickening of endometrium without gross mass, and a 4.4 cm mass in the left ovary. A sigmoidoscopy detected an invasive polypoid lesion protruding into the rectum and biopsy revealed endometriosis (Fig. 3). She decided to undergo a surgery to remove lesions and confirm the pathologic diagnosis.

**Fig. 4:**
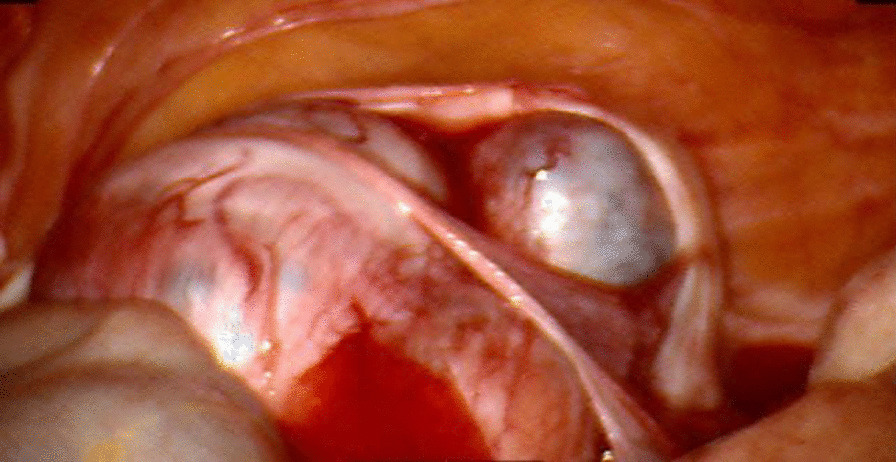
Intraoperative finding of globular lesions on the anterior uterine wall

Firstly, the vaginal protruding mass was excised completely. Secondly, the globular mass were adequately removed via laparoscopy. Thirdly, dilatation and curettage of endometrial tissue was performed. Laparoscopic findings showed 5 × 4 × 4 and 4 × 3 × 3 cm globular mass on the anterior uterine wall (Fig. 4) and a 3 × 3 × 2 cm globular mass on the rectovaginal septum; all of which were filled with chocolate-colored fluid and cheezy like materials. Histopathologically, the vaginal polypoid mass was diagnosed as endometriosis-associated complex hyperplasia and endometrioid cancer, International Federation of Gynecologic Oncology (FIGO) grade I/III. Meanwhile, globular lesions on anterior uterine wall and rectovaginal mass was diagnosed as endometriosis-associated complex hyperplasia. Furthermore, a synchronous endometrioid endometrial cancer, FIGO grade II/III was reported.

**Fig. 5:**
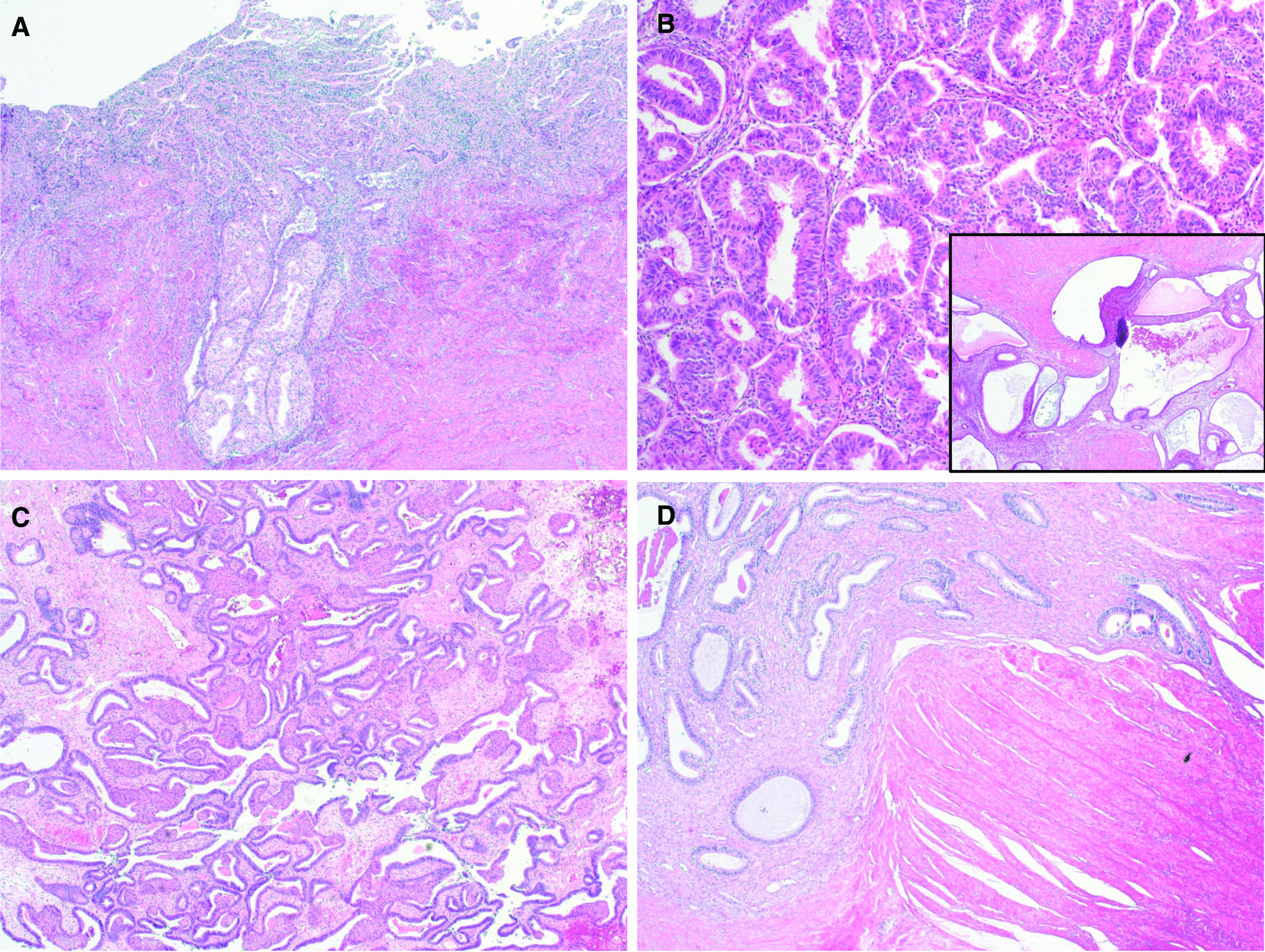
Microscopic findings of the lesion. **A** Endometrial carcinoma showing superficial myometrial invasion (X40). **B** Vaginal endometrioid carcinoma (X100) in the background of extensive endometriosis (inlet; X40). **C** Uterine serosal and parametrial wall shows complex hyperplasia in the background of endometriosis (X12). **D** Extensive endometriosis from the rectovaginal mass infiltrates rectal wall (X40)

After being confirmed as a case of synchronous vaginal and endometrial cancer, a staging surgery for endometrial cancer and en bloc extirpation of the remnant rectovaginal mass were performed. The patient underwent a total laparoscopic excision of the rectovaginal mass, radical hysterectomy and low anterior resection of the rectum. The remnant rectovaginal mass was diagnosed as only endometriosis, which invaded extensively to the rectal mucosa. Perineural invasion in the specimen of the vagina was absent. The lack of perineural invasion did not request a subsequent lymph nodal dissection [12]. The resection margin of parametria, vaginal vault, and rectum were invaded by endometriosis but, free of cancer. Finally, the patient was diagnosed as endometriosis-associated endometrioid vaginal cancer and synchronous endometrioid endometrial cancer (stage 1A1) (Fig. 5). One month post operation, she was treated with dienogest and was clinically free of disease (no evidence of disease recurrence in the imaging study) for 8 months since undergoing last surgery.

## Discussion and conclusions

Vaginal cancer constitutes 1–2% of all gynecologic malignancies. The most common histological subtype is squamous cell carcinoma, and the established risk factors include HPV infection, smoking, and decreased immune function [13]. The second most common subtype is adenocarcinoma, accounting for 15% of all primary vaginal cancer [14]. Most adenocarcinoma cases correlate with benign endometriosis [15]. In a recent comprehensive review, 37 histologically documented cases of primary vaginal cancer emerged from endometriosis [16]. In the review, 25 tumors were of epithelial histology (17 endometrioid carcinoma; 3 clear cell carcinoma; and 5 adenocarcinoma not otherwise specified), whereas 12 tumors were of sarcomatous histology.

Sampson first established the specific criteria for defining a cancer arising from endometriosis [17]: (1) the presence of benign endometrial tissue and cancer in the same site, (2) the presence of endometrial stroma surrounding the glands, and (3) the exclusion of metastasis from another primary site. According to these criteria, the vaginal mass was diagnosed as vaginal endometrioid carcinoma associated with endometriosis.

In the vaginal mass, complex hyperplasia presented synchronously with endometrioid cancer in the background of endometriosis. The step-by-step transformation process, from typical endometriosis, to atypical endometrioma, and finally to cancer, is well documented in endometriosis-associated ovarian cancer [18]. The case reveals the transition process from benign endometriosis to complex hyperplasia to endometrioid cancer in deep infiltrating endometriosis. Atypical endometriosis is considered a histologically as intermediate features between benign and malignant [19]. This term corresponds to architecturally complex and cytologically atypical proliferative lesions that resemble atypical hyperplasia arising in the endometrium. This sequential change of deep infiltrating endometriosis to malignancy has not been reported.

In this case study, rectovaginal endometriosis might have invaded the vaginal and rectal wall with a polypoid protruding appearance [20]. The main mass, located in the rectovaginal septum and cul-de-sac transformed to complex hyperplasia, whereas vaginal endometriosis transformed into endometrioid carcinoma. Meanwhile, rectal endometriosis did not develop into hyperplasia nor carcinoma. The case showed the difference in the degree of malignant change according to the organ where endometriosis is implanted; the finding was not found in the literature.

Hyperestrogenism is associated with the malignant transformation of endometriosis, and the microenvironment provided by endometriosis facilitates excess estrogen accumulation through several mechanisms [21]. Normally, aromatase is absent in a eutopic endometrial tissue, but in an endometriotic tissue, it is present in high levels [22]. This enzyme catalyzes the conversion of androstenedione and testosterone into estrone and estradiol, respectively. Its presence in endometriotic tissue leads to the constitutive expression of estradiol, and excess estradiol can result in cellular proliferation by inducing cytokine production [23]. In addition, estradiol stimulates prostaglandin E2 production, which promotes tumor growth and triggers aromatase activity, resulting in a positive feedback loop in favor of continuous estrogen formation in endometriosis [22, 24].

In obese women, the peripheral conversion of steroid hormones into estrone by aromatase increases, leading to a hyperestrogenic environment. Zanetta compared 31 patients with 62 controls and found that obese women using unopposed exogenous estrogen had a significantly increased risk of developing cancer from endometriosis (*P* = 0.05) [25]. The patient had a BMI of 36.4 kg/m^2^, and obesity-related hyperestrogenism might have been responsible for the malignant transformation of endometriosis.

Endometrial cancer is a malignant epithelial tumor that forms in the endometrium. It is the most common gynecologic malignancy in the United States [26]. The risk factors for endometrial cancer include increased estrogen levels (caused by obesity, diabetes, and high-fat diet), early age at menarche, nulliparity, late age at menopause, older age, and tamoxifen use [27–30]. Endometriosis is related to endometrial cancer, reflecting overlapping risk factors, such as endogenous or exogenous hyperestrogenism and ovulatory dysfunction [9, 10]. In a recent meta-analysis, endometrial cancer risk is 40% higher in women with endometriosis [31].

This patient was diagnosed as stage I endometriosis-associated vaginal cancer and synchronous stage IA1 endometrial cancer. The patient required the comprehensive treatment because of the combined vaginal cancer on endometriosis and endometrial cancer. Given the limited experience with endometriosis-associated vaginal carcinoma, the optimal management remains poorly established. Surgical excision of malignant lesions is employed uniformly in most patients. However, the need and composition of adjuvant therapy are not uniform and depend on patient and tumor characteristics [16]. For patients with epithelial tumors, adjuvant treatment is less frequently employed. The patient had residual endometriosis involving her vagina and rectum (resection margin with endometriosis). Medical treatment after the operation of rectovaginal endometriosis is important [32]. In the case, the progestational agent was treated to suppress any propagation of residual endometriosis.

This case demonstrated a multistep transformation from endometriosis to complex hyperplasia to endometrioid carcinoma. The progression was individualized between implantation sites and even in the same organ. Once surgery is indicated, complete excision of endometriosis lesions is needed because the remnant lesion might include the concealed malignancy.

## Data Availability

The datasets used in the current study are available from the corresponding author on reasonable request.

## Abbreviations

BMI: Body Mass Index
FIGO: International Federation of Gynecologic Oncology
